# Specific decreasing of Na^+^ channel expression on the lateral membrane of cardiomyocytes causes fatal arrhythmias in Brugada syndrome

**DOI:** 10.1038/s41598-020-76681-3

**Published:** 2020-11-17

**Authors:** Kunichika Tsumoto, Takashi Ashihara, Narumi Naito, Takao Shimamoto, Akira Amano, Yasutaka Kurata, Yoshihisa Kurachi

**Affiliations:** 1 Department of Physiology II, Kanazawa Medical University, 1-1 Daigaku, Uchinada, 920-0293 Japan; 2 Department of Pharmacology, Graduate School of Medicine, Osaka University, 2-2 Yamada-oka, Suita, 565-0871 Japan; 3 Department of Medical Informatics and Biomedical Engineering, Shiga University of Medical Science, Seta Tsukinowa-cho, Otsu, 520-2192 Japan; 4 Department of Bioinformatics, College of Life Sciences, Ritsumeikan University, 1-1-1 Nojihigashi, Kusatsu, 525-8577 Japan; 5 Glocal Center for Medical Engineering and Informatics, Osaka University, 2-2 Yamada-oka, Suita, 565-0871 Japan

**Keywords:** Arrhythmias, Computational models, Predictive medicine, Computer modelling

## Abstract

Reduced cardiac sodium (Na^+^) channel current (*I*_Na_) resulting from the loss-of-function of Na^+^ channel is a major cause of lethal arrhythmias in Brugada syndrome (BrS). Inspired by previous experimental studies which showed that in heart diseases *I*_Na_ was reduced along with expression changes in Na^+^ channel within myocytes, we hypothesized that the local decrease in *I*_Na_ caused by the alteration in Na^+^ channel expression in myocytes leads to the occurrence of phase-2 reentry, the major triggering mechanism of lethal arrhythmias in BrS. We constructed in silico human ventricular myocardial strand and ring models, and examined whether the Na^+^ channel expression changes in each myocyte cause the phase-2 reentry in BrS. Reducing Na^+^ channel expression in the lateral membrane of each myocyte caused not only the notch-and-dome but also loss-of-dome type action potentials and slowed conduction, both of which are typically observed in BrS patients. Furthermore, the selective reduction in Na^+^ channels on the lateral membrane of each myocyte together with spatial tissue heterogeneity of Na^+^ channel expression caused the phase-2 reentry and phase-2 reentry-mediated reentrant arrhythmias. Our data suggest that the BrS phenotype is strongly influenced by expression abnormalities as well as genetic abnormalities of Na^+^ channels.

## Introduction

Cardiac Na^+^ channel (Na_v_1.5) encoded by *SCN5A* that controls the excitability of cardiomyocytes mainly contributes to the action potential (AP) initiation and its propagation. Na^+^ channel dysfunction in congenital or acquired heart diseases leads to a decrease in Na^+^ channel current (*I*_Na_), which is known to be associated with slowed or blocked conduction, resulting in a potentially proarrhythmic substrate. Genetic abnormalities causing Na^+^ channel dysfunction have been linked to many arrhythmogenic diseases^1^, including sick sinus syndrome, progressive cardiac conduction disorders, and Brugada syndrome (BrS)^2^. A recent clinical study^3^ highlighted the correlation between the *I*_Na_ decrease and BrS phenotype in patients with *SCN5A* variants, which is one of the important disease genes for BrS^4^.

**Figure 1:**
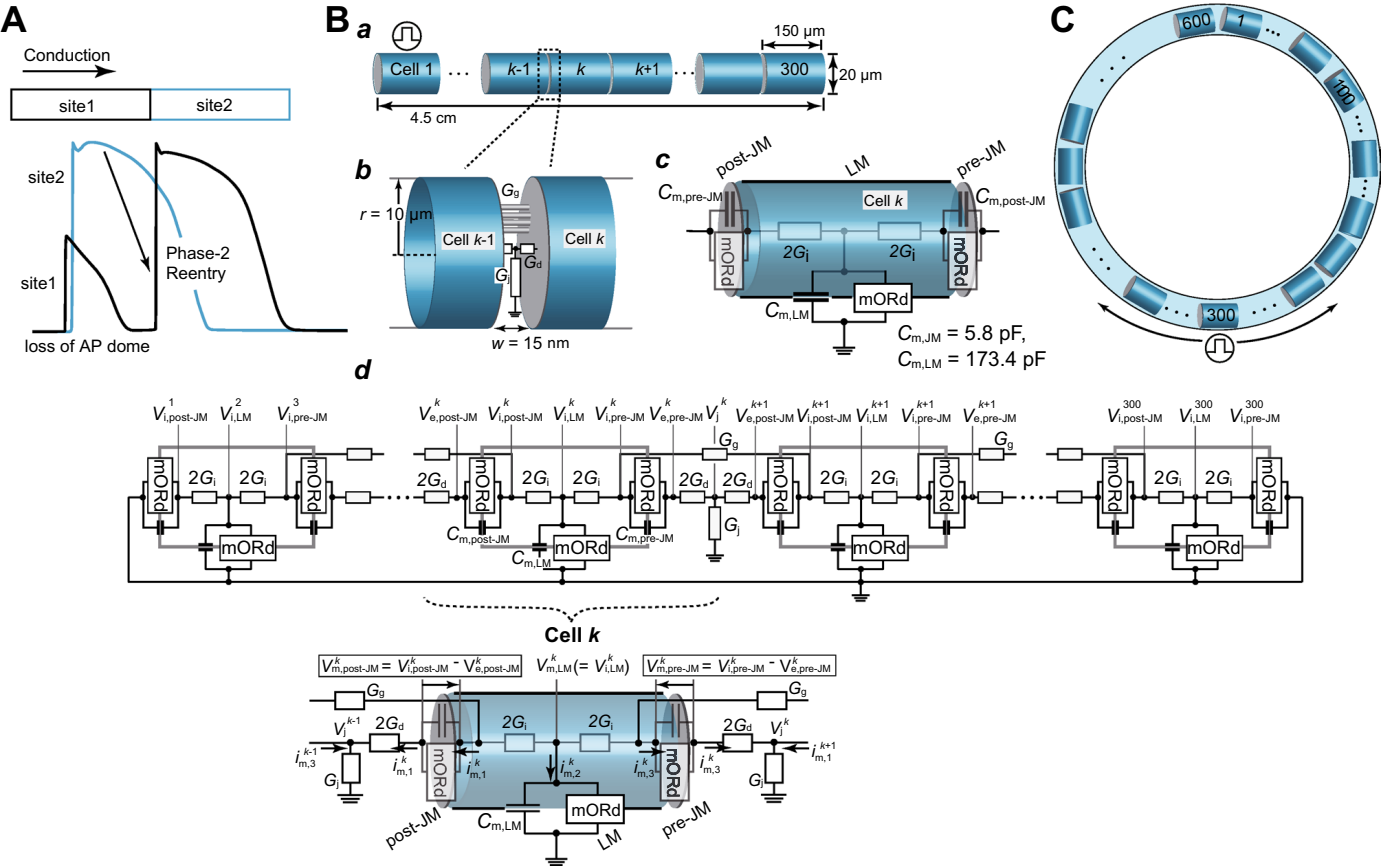
Myocardial strand and ring models. (**A**) Schematic diagram of phase-2 reentry concept. (**B**) Schematic representation of a myocardial strand model comprising cylindrical 300 cells (*a*), of intercellular junction between myocytes (*b*), of each ventricular myocyte model (*c*), and of the equivalent circuit of the myocardial strand model (d). The cell membrane in each myocyte is divided into post-junctional (post-JM), lateral (LM), and pre-junctional membrane (pre-JM) segments, and each membrane segment comprises a modified O’Hara-Rudy dynamic (mORd) ventricular myocyte model^10–12^ and membrane capacitance, *C*_m,*l*_, for *l* = post-JM, LM, and pre-JM (c). In (d), ${V}_{\text{j}}^{k}$ represents extracellular cleft potential just after the *k*th myocyte. ${V}_{\text{i},l}^{k}$, and ${V}_{\text{e},l}^{k}$ , for *l* = post-JM, LM, and pre-JM, indicate the intracellular and extracellular potentials, respectively, of *l*th segment of the *k*th myocyte. ${V}_{\text{m}, l}^{k}$ denotes the transmembrane potential, i.e., ${V}_{\text{m},l}^{k}={V}_{\text{i},l}^{k}-{V}_{\text{e},l}^{k}$. (**C**) Schematic representations of the myocardial ring model comprising 600 cells. Cell #1 (**B**) and #300 (**C**) were electrically stimulated at 1 Hz. *G*_g_, gap junction conductance; *G*_j_, radial conductance of intercellular cleft; *G*_d_, axial conductance of intercellular cleft.

BrS is characterized by the electrocardiograms with both a right bundle-branch block pattern and ST-segment elevation in the right precordial leads (V_1_–V_3_) and by the higher incidence of sudden cardiac death due to ventricular tachycardia/fibrillation (VT/VF)^5,6^. It is commonly believed that VT/VF is elicited by the closely coupled premature ventricular contractions via the phase-2 reentry (P2R) mechanism^6^. This mechanism is that APs with phase-2 dome in some sites of the tissue re-excite neighboring repolarized sites via electrotonic interaction to evoke an additional conductive AP (triggered activity) (Fig. 1A). Although at first, it has been predicted that a global difference in AP duration (APD) causing a transmural voltage gradient in the right ventricle (RV) is a cause of P2R development^7^, several experimental studies using an optical mapping system showed that the epicardial local APD difference was involved in the occurrence of P2R^8,9^. Thus, the P2R mechanism in BrS is still controversial.

We previously showed that subcellular alterations in Na^+^ channel expression induced by myocardial ischemia, specifically reduced number of Na^+^ channels in the lateral membrane (LM) of cardiomyocytes, was strongly associated with both the decrease in local *I*_Na_ and slowed conduction^13^. Furthermore, the local decreasing of *I*_Na_ in cardiomyocytes led to the development of P2R^13^. Therefore, we hypothesized that the developments of P2R and VT/VF in BrS are brought about by local alterations in subcellular Na^+^ channel expression. To validate this hypothesis, we performed simulations of AP propagation in the in silico models and investigated the relationship between the subcellular alterations in Na^+^ channel expression and the occurrence of P2R, and whether the P2R leads to reentrant arrhythmia onsets in BrS.

## Results

### Reducing Na^+^ channel conductance in the LM causes AP morphological changes

Experiments on knock-in mice lacking the Serine-Isoleucine-Valine domain (∆SIV) of the Na_v_1.5 PDZ domain-binding motif have demonstrated that both *I*_Na_ and Na^+^ channel expression on the LM, but not on the intercalated discs (IDs), of cardiomyocytes were decreased by the lack of ∆SIV in Na^+^ channels^14,15^. Therefore, we first examined effects of selective decreasing of Na^+^ channel expression on the LM of cardiomyocytes on AP propagation by using a homogeneous myocardial strand model consisting of 300 cells (see “Methods” and Supplementary Methods; Fig. 1B). Throughout the article, we express Na^+^ channel conductances of the junctional membrane (JM), i.e. ID, and LM as percentages of the control conductance (see “Methods”), i.e., %*g*_Na,JM_ and %*g*_Na,LM_, respectively.

**Figure 2:**
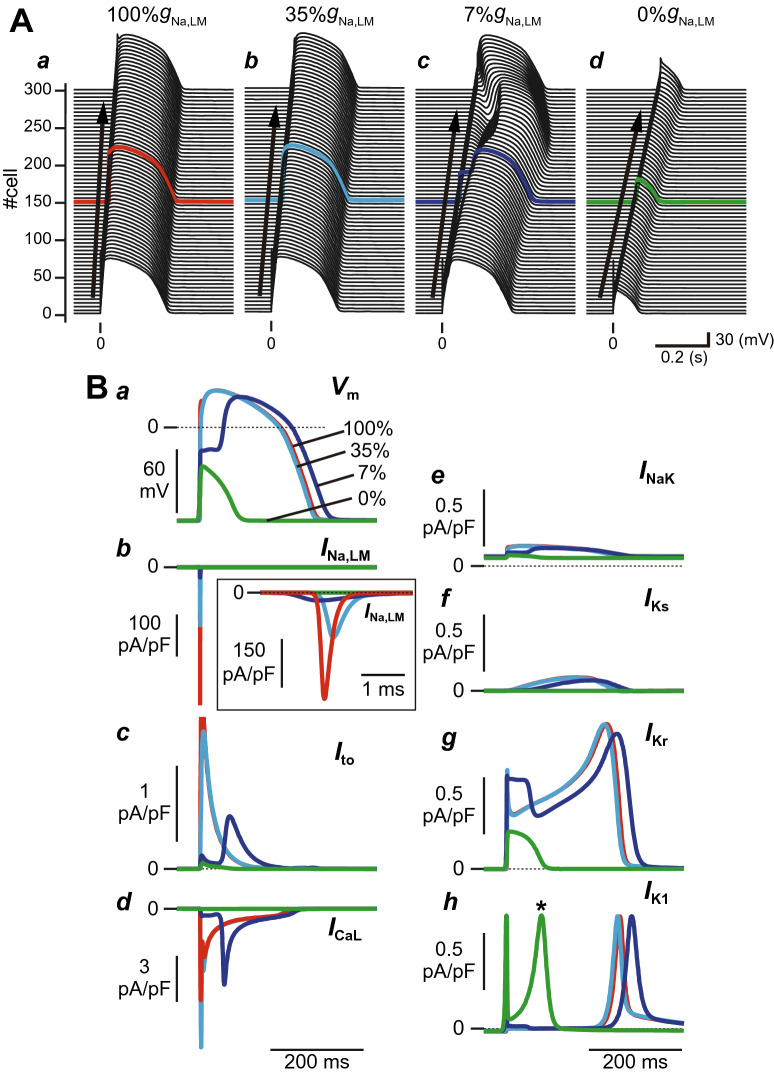

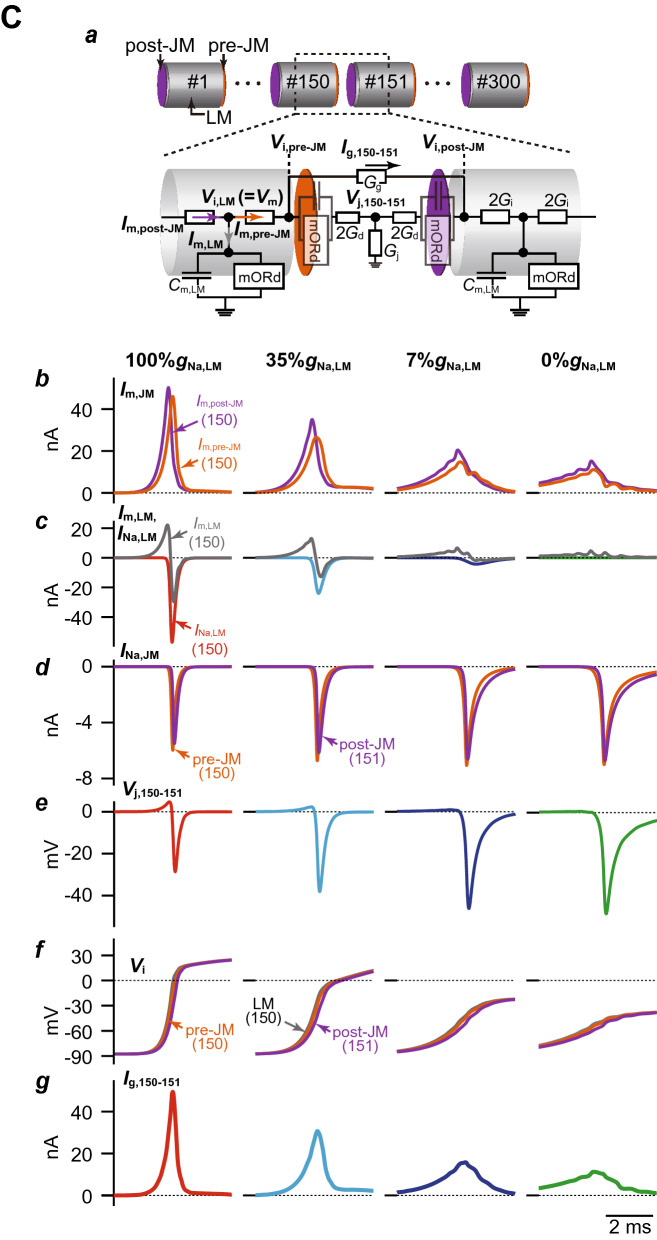
Effects of changes in the subcellular Na^+^ channel expression on action potential (AP) morphology and conduction velocity (CV). (**A**) AP propagation observed in the myocardial strand model with a spatially-homogeneous reduction in Na^+^ channel conductance in the lateral membrane (LM) of 100%*g*_Na,LM_ (*a*), 35%*g*_Na,LM_ (*b*), 7%*g*_Na,LM_ (*c*), and 0%*g*_Na,LM_ (*d*). (**B**) AP morphological changes (*a*), *I*_Na_ at the LM, *I*_Na,LM_ (*b*), *I*_to_ (*c*), *I*_CaL_ (*d*), *I*_NaK_ (*e*), *I*_Ks_ (*f*), *I*_Kr_ (*g*), and *I*_K1_ (*h*) at the LM. (**C**) Schematic diagram of a intercellular junction part in the myocardial strand model (*a*), the post-junctional (*I*_m,post-JM_) and pre-junctional transmembrane currents (*I*_m,pre-JM_) (*b*), the transmembrane current (*I*_m,LM_) and *I*_Na_ in the LM segments (*c*), the *I*_Na,JM_ of the pre-junctional membrane (pre-JM) in the 150th myocyte and of the post-junctional membrane (post-JM) in the 151st myocyte (*d*), the extracellular cleft potential (*V*_j_) between the 150th and 151st myocytes (*e*), the intracellular potential (*V*_i_) of the LM and pre-JM in 150th myocyte and of the post-JM in 151st myocyte (*f*), and the gap junctional current (*I*_g_) between the 150th and 151st myocytes (*g*). The APs and ion currents were recorded in a myocyte located at the middle of the myocardial strand (cell #150). Each AP morphology corresponds to the waveforms in panel (**A**) indicated by red, cyan, blue, and green lines. CVs at 100%*g*_Na,LM_, 35%*g*_Na,LM_, 7%*g*_Na,LM_ and 0%*g*_Na,LM_ were 71.4, 53.6, 33.3, and 25.0 cm/s, respectively (see Table 1).

When the Na^+^ channel conductance in each myocyte of the myocardial fiber was set to the control condition (100%*g*_Na,JM_ and 100%*g*_Na,LM_), the APs propagated through the myocardial strand (Fig. 2A*a*) and exhibited a typical human ventricular AP morphology (Fig. 2A*a*,B*a*, red line). Reducing the %g_Na,LM_ by 65%, corresponding to 51.3% of the total Na^+^ channel conductance (%*g*_Na,tot_) per myocyte, resulted in the decrease in the *I*_Na,LM_ peak in the 150th cell by 57.9% (Fig. 2B*b*, cyan line, and Table 1) and slightly shortened the APD; APD at 90% repolarization (APD_90_) was 236.9 ms in 35%*g*_Na,LM_ versus 241.8 ms in 100%*g*_Na,LM_ (Fig. 2A*b*,B*a*, and Table 1). These results were similar to those experimentally determined by Shy et al.^15^ (see Supplementary Table S1**)**. As the %g_Na,LM_ further decreased step-by-step (7%*g*_Na,LM_; 26.6%*g*_Na,tot_), AP phase-0 amplitude decreased, and a larger phase-1 dip in the AP was produced (Fig. 2A*c*,B*a*, blue lines). As a result, the peak of the phase-2 dome was markedly delayed. APD_90_ with delayed phase-2 dome peak was 26.9 ms longer than that of the control condition (100%*g*_Na,LM_, see Table 1). Moreover, the loss of Na^+^ channels in the LM of each myocyte (0%*g*_Na,LM_; 21.0%*g*_Na,tot_) caused a further decrease in the AP phase-0 amplitude (Fig. 2B*a*, green line), resulting in the failure of the transient outward K^+^ channel current (*I*_to_) and L-type Ca^2+^ channel current (*I*_CaL_) to activate normally (Figs. 2B*c*,*d*, green lines). The transient activation of the rapid component of delayed rectifier K^+^ channel current (*I*_Kr_) and the Na^+^-K^+^ pump current (*I*_NaK_), and subsequent activation of the inward rectifier K^+^ channel current (*I*_K1_), the late peak of which appeared earlier, allowed for earlier repolarization (Figs. 2B*e*,*g*,*h*). The slow component of delayed rectifier K^+^ channel current (*I*_Ks_) hardly contributed to the repolarization (Fig. 2B*f*, green line). This, in turn, led to AP propagation with a shortened APD (Fig. 2A*d*).

**Table 1:** Effects of changes in Na^+^ channel density of the lateral membrane on epicardial action potential parameters, the regional Na^+^ channel current, and conduction velocity.

	100%*g*_Na,LM_	35%*g*_Na,LM_	7%*g*_Na,LM_	0%*g*_Na,LM_
APD_90_ (ms)	241.8	236.9	268.7	80.9
${\dot{{\varvec{V}}}}_{\mathbf{m}\mathbf{a}\mathbf{x}}$(mV/ms)	238.6	96.0	39.0	26.5
RMP (mV)	− 87.7	− 87.7	− 87.7	− 87.8
AAP (mV)	122.6	122.4	116.5	51.2
Peak *I*_Na,LM_ (pA/pF)	329.0	138.5	24.6	0.0
Peak post-*I*_Na,JM_ (pA/pF)	951.7	1064.9	1143.9	1162.4
Peak pre-*I*_Na,JM_ (pA/pF)	1035.0	1166.5	1222.8	1211.8
CV (cm/s)	71.4	53.6	33.3	25.0

Each parameter is determined for the LM segment of the 150th cell in the myocardial strand model comprised of 300 epicardial ventricular myocytes.

*%g*_Na,LM_ a percent *g*_Na_ in the lateral membrane (LM) segment of each cell of the strand model; APD_90_ action potential duration measured at 90% repolarization; ${\dot{V}}_{\text{max}}$, maximum upstroke velocity of action potential; RMP, resting membrane potential; AAP, amplitude of action potential; Peak *I*_Na,LM_, post-*I*_Na,JM_ , and pre-*I*_Na,JM_, the respective peak values of *I*_Na_ across the LM, post-JM, and pre-JM segments of the 150th cell in the myocardial strand model; CV, conduction velocity.

Furthermore, we examined effects of selective decreasing of Na^+^ channel expression on the JMs of cardiomyocytes (Supplementary Fig. S1), and of uniform decreasing of Na^+^ channel expression on both JMs and LM on AP propagation (Supplementary Fig. S2). As shown in Supplementary Fig. S1, the selective decreasing of Na^+^ channels on the JMs did not affect AP morphology. On the other hand, the uniform decreasing of the Na^+^ channels on both JMs and LM to 10% or less resulted in the AP morphology changes characterized by the loss-of-dome (see Supplementary Fig. S2). These results suggest that changes in Na^+^ channel expression on the LM have a greater effect on AP morphology than those on the JMs.

These alterations in subcellular Na^+^ channel distribution also modified the conduction velocity (CV). The pre-JM current (*I*_m,pre-JM_; Fig. 2C*a*,*b*, orange lines) is the difference between the post-JM current (*I*_m,post-JM_; Fig. 2C*a*,*b*, purple lines) and the transmembrane current in the LM (see Fig. 2C*c*, gray lines), i.e., *I*_m,pre-JM_ = *I*_m,post-JM_ − *I*_m,LM_, and is determined by *G*_i_ and *V*_i,LM_ − *V*_i,pre-JM_, where *V*_i,LM_ and *V*_i,pre-JM_ are the intracellular potentials in the LM and pre-JM compartments, respectively (see Fig. 2C*a*,*f*). The decrease in Na^+^ channel expression on the LM (%*g*_Na,LM_ from 100 to 0%; see Fig. 2C*c*) led to not only a decrease in the *I*_Na,LM_ but also a slight increase in the *I*_Na,JM_ due to excessive localization of Na^+^ channels to the JMs (see Fig. 2C*d*), consequently resulting in a large negative cleft potential (*V*_j_) at each intercellular junctions, corresponding to augmentation of the ephaptic interaction (see Fig. 2C*e*). This decrease in *I*_Na,LM_ also caused a decrease in the maximum upstroke velocity of *V*_i,LM_ , i.e., ${\dot{V}}_{\text{max}}$ (see Fig. 2C*f*, gray lines and see Table 1). Because the decrease in ${\dot{V}}_{\text{max}}$ led to a decrease in the difference between the *V*_i,LM_ and *V*_i,pre-JM_ (Fig. 2C*f*, gray and orange lines), the decrease in *I*_Na,LM_ also reduced *I*_m,pre-JM_ (compare orange lines in left to right panels of Fig. 2C*b*), resulting in the decrease in maximum upstroke velocity of *V*_i,pre-JM_, ${\dot{V}}_{\text{i},\text{pre-JM}}^{\text{max}}$, (compare orange lines in left to right panels of Fig. 2C*f*). Thus, the decrease in ${\dot{V}}_{\text{i},\text{pre-JM}}^{\text{max}}$ led to a decrease in the difference between the *V*_i,pre-JM_ and the intracellular potential of the post-JM compartment in the neighbor myocyte (*V*_i,post-JM_; purple lines in Fig. 2C*f*). Subsequently, this caused both a marked decrease in the gap junctional current (*I*_g_) defined by the equation ${I}_{\text{g}}={G}_{\text{g}}\times \left({V}_{\text{i},\text{pre-JM}}^{150}-{V}_{\text{i},\text{post-JM}}^{151}\right)$ for 150th myocyte (compare colored lines in left to right panels of Fig. 2C*g*) and a decrease in CV (see Table 1).

### Combination of the decreases in Na^+^ channel expression on the LM in myocytes and the spatially-heterogeneous Na^+^ channel distribution in tissue led to P2R development

**Figure 3:**
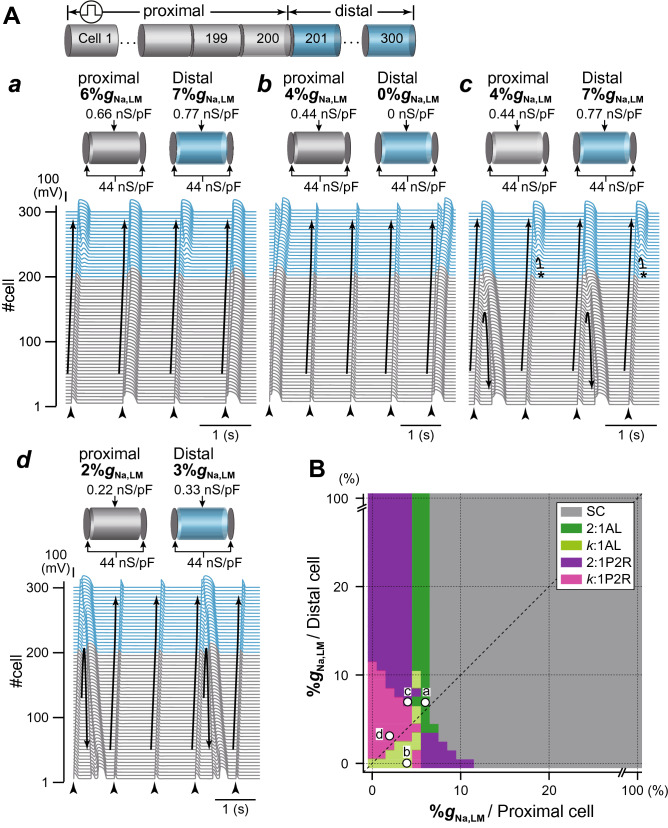
Simulated phase-2 reentry (P2R) development in the strand model. (**A**) Examples of typical patterns of AP propagation observed in the myocardial strand model with a spatially-heterogeneous Na^+^ channel distribution. The Na^+^ channel conductances on the LM in the proximal (cell #1–200) and the distal (cell #201–300) were set to 6%*g*_Na,LM_ and 7%*g*_Na,LM_, respectively, to cause 2:1 AP alternans (*a*), 4%*g*_Na,LM_ and 0%*g*_Na,LM_, respectively, to cause 4:1 AP alternans (*b*), 4%*g*_Na,LM_ and 7%*g*_Na,LM_, respectively, to cause 2:1 retrograde P2R (*c*), and 2%*g*_Na,LM_ and 3%*g*_Na,LM_, respectively, to cause 3:1 P2R consisting of a retrograde P2R and twice conductions with abbreviated AP (*d*). Arrows and black short bars indicate the direction of AP propagation and blockage, respectively. (**B**) A phase diagram of AP propagation patterns for the %*g*_Na,LM_ in proximal myocytes vs. %*g*_Na,LM_ in distal myocytes. Open circles labeled as *a*–*d* correspond to the parameter sets at which the AP propagation patterns shown in panels **A***a*–*d*. SC, stable conduction consisting of typical AP morphology of human ventricular myocytes; 2:1 AL, 2:1 AP alternans pattern; *k*:1 AL, AP alternans pattern including a typical AP conduction and *k* − 1 times conduction with abbreviated AP; 2:1 P2R, 2:1 alternans pattern including a retrograde phase-2 reentry and an abbreviated AP conduction; *k*:1 P2R, *k*:1 alternans pattern including a retrograde phase-2 reentry and *k* − 1 times conduction with abbreviated AP.

To test our hypothesis that alterations in the subcellular Na^+^ channel distribution lead to the development of P2R, we investigated the combined effects of both the regional *I*_Na_ decrease on the LM in myocytes and the spatial heterogeneity of Na^+^ channels in the myocardial tissue on AP propagation. The spatial heterogeneity of Na^+^ channels in the myocardial strand was achieved by changing the %*g*_Na,LM_ in the proximal part of the myocardial strand (200 myocytes), while maintaining the 7%*g*_Na,LM_ (26.6%*g*_Na,tot_) in the distal part of the myocardial strand (100 myocytes). The AP conduction exhibited AP alternans (Fig. 3A*a*) when the %*g*_Na,LM_ of the proximal myocytes was set to 6%*g*_Na,LM_ (25.8%*g*_Na,tot_), as the slight spatial heterogeneity of Na^+^ channels in the myocardial strand. The ionic mechanism underlying AP alternans is shown in Supplementary Fig. S3. When the APD was prolonged due to reduced phase-0 amplitude and delayed phase-2 dome formation (e.g., green trace in Supplementary Fig. S3A), the recovery from the inactivation state of Na^+^ channels in the LM slightly delayed compared to that in the case of loss-of-dome (compare the blue and green traces in Supplementary Fig. S3C), slightly decreasing the peak *I*_Na,LM_ to cause the loss-of-dome at the next excitation (see Supplementary Fig. S3B–D). Consequently, the AP phase-0 amplitude slightly decreased (blue trace in Supplementary Fig. S3A), and the *I*_CaL_ failed to activate (blue trace in Supplementary Fig. S3E). The failure of *I*_CaL_ activation led to shortening of APD and prolongation of the diastolic interval (DI). The longer DI augmented the recovery from the inactivation state of the Na^+^ channel in the LM segment, causing the increase in the peak *I*_Na,LM_ (see green trace in Supplementary Fig. S3D) followed by the full AP accompanied by the delayed phase-2 dome (see green trace in Supplementary Fig. S3A). In addition, depending on the spatial heterogeneity of Na^+^ channels in the myocardial tissue, the AP propagation exhibited complex alternans patterns consisting of *k* − 1 times abbreviated AP conduction and a typical AP conduction (*k*:1 alternans) or a retrograde P2R (*k*:1 P2R). Figure 3A*b*–*d* show examples of 4:1 AP alternans, 2:1 P2R, and 3:1 P2R, respectively.

To understand relationships among Na^+^ channel distributions within myocytes, spatial heterogeneous Na^+^ channel distribution in tissue, and development of P2R, we systematically performed simulations of AP propagations while changing the subcellular Na^+^ channel distribution in the proximal and distal parts of the myocardial fiber every 1%*g*_Na,LM_. Figure 3B shows a phase diagram constructed by mapping the AP propagation patterns obtained from each simulation on the parameter plane of the %*g*_Na,LM_ in proximal and distal myocytes. We can see that the 2:1 retrograde P2R as shown in Fig. 3A*b* occurred when the %*g*_Na,LM_ in the proximal myocyte was reduced to < 5% regardless of the %*g*_Na,LM_ in the distal myocytes (Fig. 3B, purple region), and that the region where P2R occurs was largely the left upper region of the diagonal line which represents conditions of spatially-homogeneous Na^+^ channel distribution in myocardial strand. This implies that P2R occurs when a spatially-heterogeneous Na^+^ channel distribution exists on the myocardial fiber. These results suggest that selective decrease in Na^+^ channels on the LM of myocytes and the spatially-heterogeneous Na^+^ channel expression on the myocardial tissue cooperatively facilitate the occurrence of P2R.

In addition, we examined the effect of cleft width (*cw*) changes on the P2R development because AP propagation with both gap-junctional and ephaptic interacted mechanisms (see “Methods”) is greatly sensitive to the *cw*^16,17^. When the *cw* was in the range of 7 to 17 nm, the occurrence of P2R was confirmed (Supplementary Fig. S4). On the other hand, with *cw* < 7 nm, the ephaptic interaction, i.e., the cleft potential (*V*_j_) at intercellular junctions, was significantly augmented because of an increase in the cleft resistance (see Fig. 1B, “Methods”) due to the decrease in *cw*. The *V*_j_ amplitude increase speeded up the AP depolarization in the JMs and reduced the potential differences between the pre- and post-JMs, leading to the gap-junctional current *I*_g_. The *I*_g_ decrease caused AP propagation to fail. On the contrary, as the *cw* increases to more than 17 nm, the diminishing *V*_j_ (compare each panel *c* in Supplementary Fig. S4A–D, left to right) led to the relative increases in the local current consisting of the *I*_g_ and *I*_Na,JM_ (see panels *d*-*h* in Supplementary Fig. S4A–D). Thereby, the loss-of-dome AP observed in the proximal myocytes with smaller *cw* changed to a typical ventricular AP morphology (compare each panel *b* in Supplementary Fig. S4B,C). Furthermore, in the case of the gap-junctional mechanism alone corresponding to the loss of the *V*_j_ (no ephaptic coupling), the P2R did not occur, causing the normal AP conduction (see Supplementary Fig. S4D). These results suggest that ephaptic interactions are essential to the occurrences of P2R in patients with BrS.

### I_CaL_ plays an important role in the P2R development

**Figure 4:**
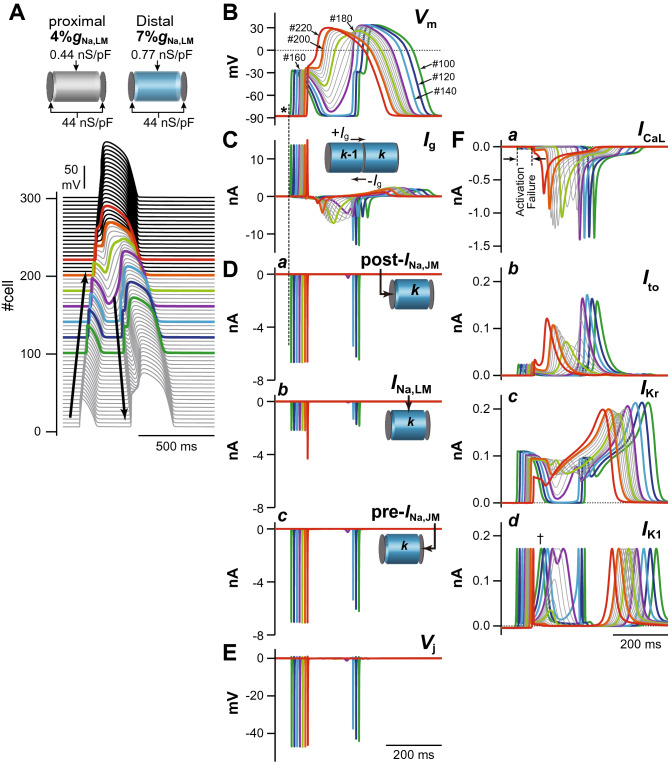
Mechanism of phase-2 reentry (P2R). (**A**) The simulated AP propagation in the myocardial strand model during the development of 2:1 P2R shown in Fig. 3A*b*. (**B**) Simulated behaviors of the membrane potential (*V*_m_) in several myocytes (#100–220) during the 2:1 P2R development. (**C**) The gap junctional currents, *I*_g_, flowing from the cell #100 to #220. (**D**) *I*_Na_ in the post-junctional membrane (*I*_Na,post-JM_) (*a*), the lateral membrane (*I*_Na,LM_) (*b*), and the pre-junctional membrane (*I*_Na,pre-JM_) (*c*). (**E**) The extracellular cleft potential, *V*_j_, in the cell #100 to #220. **F**, *I*_CaL_ (*a*), *I*_to_ (*b*), *I*_Kr_ (*c*), and *I*_K1_ (*d*) during the P2R development.

Based on the results shown in Fig. 3, we focused on how AP morphology is affected by changes in the ion channel currents. Figure 4 shows the AP profiles and main ion channel currents in several myocytes near the border between the proximal and distal parts of the myocardial fiber during the development of the 2:1 P2R shown in Fig. 3A*b*. The initial depolarization of the regional membrane potential (*V*_m_) in the LM segment of each myocyte, i.e., that is equal to *V*_i,LM_, within the proximal part of the myocardial strand (#100, 120 and 140 in Fig. 4B, asterisk) was formed by the local current flows consisting of *I*_g_ (Fig. 4C) and post-*I*_Na,JM_ (Fig. 4D*a*). These current flows entered the myocyte through the post-JM, and depolarized the LM and pre-JM, resulting in the *I*_Na,LM_ (Fig. 4D*b*) and pre-*I*_Na,JM_ (Fig. 4D*c*). Furthermore, these post- and pre-*I*_Na,JM_ led to the large negative cleft potential (*V*_j_) at each intercellular junction (Fig. 4E). However, the local current was not able to depolarize the LM sufficiently (Fig. 4B), and both *I*_CaL_ and *I*_to_ failed to activate (Fig. 4F*a*,*b*). Moreover, the relatively large transient *I*_Kr_ activation (Fig. 4F*c*) and faster onset of the late peak of *I*_K1_ in the proximal myocyte (Fig. 4F*d*, dagger) accelerated the AP repolarization, leading to the loss of AP dome (Fig. 4A,B). Subsequently, the difference in *V*_m_ at the border between proximal and distal parts was increased, and consequently this elicited the retrograde *I*_g_ (the inward currents in Fig. 4C) from the distal to the proximal part. Unlike the anterograde *I*_g_ elicited by the initial depolarization differences in neighbor myocytes within the proximal part, the retrograde *I*_g_ continued to flow for a relatively long time because the notch-and-dome type prolonged AP in distal myocytes caused a long-lasting potential difference from the proximal loss-of-dome abbreviated AP. Continuous depolarizing loads of the proximal myocytes by the long-lasting retrograde *I*_g_ allow the depolarization of the *V*_m_ that activates *I*_CaL_ of the LM in proximal myocytes, leading to re-excitation of the myocytes in the proximal part, i.e., retrograde P2R (Fig. 4A).

**Figure 5:**
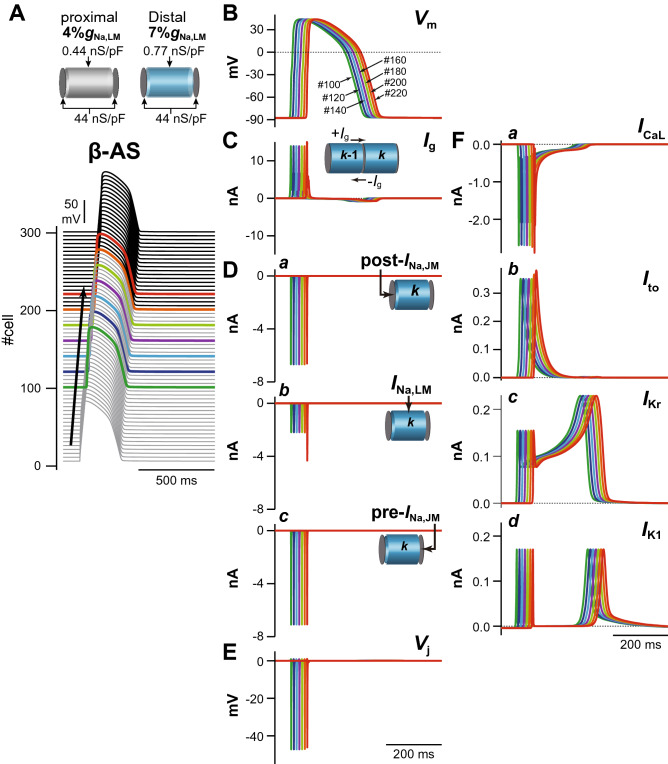
Mechanisms for the prevention of phase-2 reentry (P2R) under *β*-AS condition. (**A**,**B**) The simulated AP propagation (**A**) and the membrane potential (*V*_m_) change (**B**) of several myocytes in the myocardial strand model under the same condition as for Fig. 3A*b* but during *β*-AS. (**C**–**F**) The gap junctional current, *I*_g_ (**C**), *I*_Na_ in the post-junctional membrane (JM), *I*_Na,post-JM_ (**D***a*), *I*_Na_ in the lateral membrane, *I*_Na,LM_ (**D***b*), and *I*_Na_ in the pre-JM, *I*_Na,pre-JM_ (**D***c*), The extracellular cleft potentials, *V*_j_ (**E**), *I*_CaL_ (**F***a*), *I*_to_ (**F***b*), *I*_Kr_ (**F***c*), and *I*_K1_ (**F***d*) during *β*-AS.

This theoretical model for the P2R mechanism can also be used to investigate the mechanism of P2R inhibition. We conducted an additional, identical simulation under a *β*-AS condition^18,19^. The marked increase in *I*_CaL_ due to the *β*-AS effect kept the AP dome of each myocyte even under the smaller %*g*_Na,LM_ condition that caused loss-of-dome type abbreviated AP by the marked decrease in *I*_Na,LM_ in the proximal part of the myocardial fiber without *β*-AS (see Fig. 5). This resulted in an increase in *V*_m_ amplitude and APD prolongation in the proximal and distal parts of the myofiber (compare respective panels in Figs. 4 and 5). On the other hand, as shown in Supplementary Fig. S5, the P2R development could also be suppressed by reducing *I*_CaL_. Thus, not only *β*-AS that causes an increase in *I*_CaL_ but also *β*-blocker may be effective for preventing P2R.

### The P2R triggered reentrant arrhythmias

**Figure 6:**
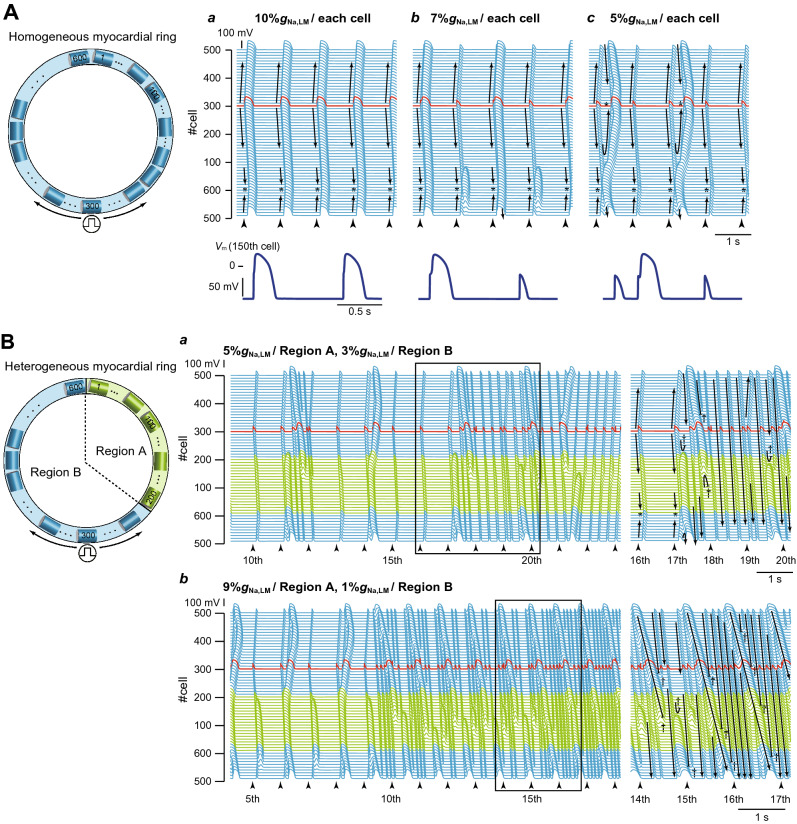

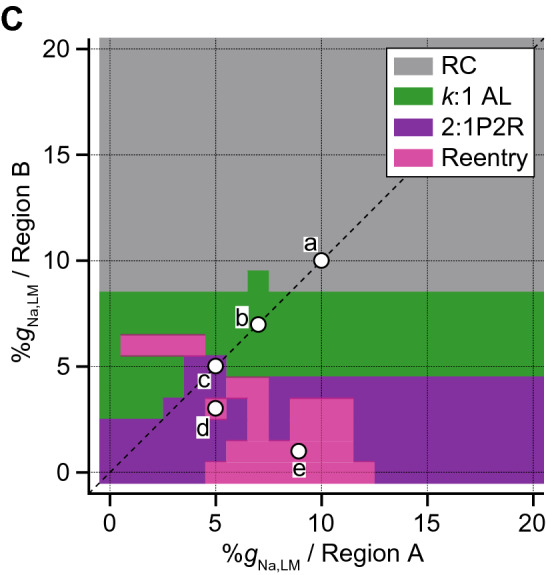
Rotatory reentry induction in the myocardial ring model. (**A**) Overall views (top) of simulated AP propagations in the myocardial ring with spatially-homogeneous Na^+^ channel distribution with 10%*g*_Na,LM_ (*a*), 7%*g*_Na,LM_ (*b*), and 5%*g*_Na,LM_ (*c*) in the lateral membrane (LM) segment of each myocyte and enlarged views (bottom) of the time-dependent behaviours of membrane potential (*V*_m_) of the 150th myocyte. (**B**) Overall (*left*) and enlarged views (*right*) of simulated AP propagations in the same model but with a spatially-heterogeneous Na^+^ channel distribution with *g*_Na,LM_ in the LM of the 1st to 200th myocytes (Region A) and of the 201st to 600th myocytes (Region B): 5%*g*_Na,LM_ in Region A and 3%*g*_Na,LM_ in Region B (*a*) and 9%*g*_Na,LM_ in Region A and 1%*g*_Na,LM_ in Region B (*b*). Arrows and black short bars indicate the direction of AP propagation and blockage, respectively. Asterisks and dagger symbols represent the collision of excitation wavefronts, and the blockade of AP propagation, respectively. The red trace represents the AP behavior at the stimulus site (cell #300). (**C**) A phase diagram of AP propagation patterns for the %*g*_Na,LM_ of each myocyte within Region A vs. %*g*_Na,LM_ of each myocyte within Region B. Open circles labeled as *a*–*c* correspond to the parameter sets at which the AP propagation patterns shown in **A***a*–*c* occurred. Open circles labeled as (*d*) and (*e*) show the parameter sets for (**B***a*,*b*).

Whether or not the P2R triggers reentrant arrhythmias was investigated using a myocardial ring model (Fig. 1C). In the myocardial ring with spatially-homogeneous Na^+^ channel distribution (Fig. 6A), the AP exhibited bidirectional conduction from the stimulus site (300th myocyte), resulting in the collision of excitation wavefronts at the opposite side of the stimulus site (Fig. 6A*a*, asterisk). When the %*g*_Na,LM_ of each myocyte in the myocardial ring was set to 7%, we could observe the bidirectional conduction with 2:1 alternans pattern such that the loss-of-dome AP and the AP with decreased AP phase-0 amplitude and delayed phase-2 dome formation appear alternately (see Fig. 6A*b*). When the %*g*_Na,LM_ in the LM of each myocyte in the myocardial ring was homogeneously reduced by 95%, the bidirectional conduction of shortened AP from the stimulus site was observed (Fig. 6A*c*); the bidirectional excitation wavefronts collided, causing a bidirectional P2R in the myocardial ring. However, each excitation wavefront originating from the bidirectional P2R collided and disappeared at around stimulus site (Fig. 6A*c*); no proarrhythmic changes were observed in the myocardial ring model with spatially-homogeneous Na^+^ channel distribution even under the condition of the markedly decreased %*g*_Na,LM_. However, in the same myocardial ring model but with the spatially-heterogeneous Na^+^ channel distribution (Fig. 6B), AP conduction properties obviously changed (see Fig. 6B*a*,*b*). With setting the %*g*_Na,LM_ of 200 cells (cells #1–200; region A in Fig. 6B) to 5% (0.55 nS/pF), the %*g*_Na,LM_ in each 400 cells (cells #201–600; region B in Fig. 6B) was uniformly reduced to 3% (0.33 nS/pF), thereby producing the spatial heterogeneity of Na^+^ channel distribution within the tissue. The bidirectional excitation wavefronts elicited at the stimulus site (cell #300) collided at the border between the regions A and B (asterisk in the right panel of Fig. 6B*a*) and were subsequently followed by bidirectional P2R occurrence. However, the clockwise-rotating excitation wave of the P2R exhibited decremental conduction and block (dagger in the right panel of Fig. 6B*a*); in contrast, the counterclockwise-rotating excitation wave continued to conduct in the myocardial ring, causing the P2R-mediated reentrant tachyarrhythmia. During 1-Hz pacing, the occurrence of reentrant arrhythmias was intermittent and irregular (see Fig. 6B*a*, *left*). The further augmentation of the spatial heterogeneity of Na^+^ channel distribution in the tissue (9%*g*_Na,LM_ in region A and 1%*g*_Na,LM_ in region B on the myocardial ring) induced P2R-mediated persistent reentry (Fig. 6B*b*). Notably, a reentrant wave with a large amplitude slowly rotated on the myocardial ring, while continuously emitting fast but low-amplitude waves. This low-amplitude fast wave circled around and collided with the tail of the slow wave (Fig. 6B*b*). To know what degree of the spatial heterogeneity in %*g*_Na,LM_ for the two regions in the myocardial ring is needed to cause reentrant arrhythmias, we constructed a phase diagram of the AP conduction properties (see Fig. 6C). This result indicates that the P2R-mediated reentry occurred mostly when the %*g*_Na,LM_ in the region B was reduced to < 5% and the %*g*_Na,LM_ in the region A was in the 5 ~ 10% range (Fig. 6C, magenta region).

## Discussion

Although it has so far believed that in addition to the loss-of-function mutation of Na^+^ channels, impaired Na_v_1.5 trafficking^20–23^ or Na^+^ channel expression abnormality^14,15^ to the cell surface membrane is one of the major causes of fatal arrhythmias in patients with BrS, the link between the loss-of-function of Na^+^ channels and arrhythmogenesis was not clear. In the present study, we found that selective decreases in *I*_Na,LM_ of each ventricular myocyte without changing *I*_Na,JM_ (Na^+^ channel located at junctional membranes) caused both morphological changes in AP and the conduction slowing that are typically observed in BrS patients^24,25^. These findings suggest that both the BrS mechanisms, previously represented as repolarization and depolarization abnormalities^26^, can at least in part be explained by this decrease in *I*_Na,LM_. Furthermore, we showed that the marked decrease in *I*_Na,LM_ together with an existence of the spatial heterogeneity at tissue-level in the Na^+^ channel expression is essential for the P2R development, as well as the reentry initiation triggered by the P2R. To the best of our knowledge, this is the first report demonstrating mechanistic links between the *I*_Na_ decrease as the subcellular Na^+^ channel expression changes and P2R in BrS.

Selectively reducing Na^+^ channels from the LM led to the notch-and-dome AP morphology (Fig. 2A*c*,B, blue lines). The underdeveloped AP phase-0 amplitude resulted in a marked decrease in *I*_to_ during AP phase-1 and the delay of *I*_CaL_ activation because both *I*_to_ and *I*_CaL_ activate as *V*_m_ exceeds − 30 mV^10^. The decrease in *I*_to_ and delay in *I*_CaL_ activation caused a large phase-1 dip and the delay of the AP phase-2 dome peak, respectively. This AP shape is greatly similar to those reported in our previous studies^13,16^, and in Wei et al.^17^, which has shown the occurrence of AP with the delayed phase-2 dome by changing the cleft width at the intercellular junction. There is a common mechanism for APs generated in those models, and the success or failure of L-type Ca^2+^ channel activation determines whether an AP exhibits the delayed phase-2 dome or the loss-of-dome. This morphology resembled that of epicardial monophasic AP recordings from the RV outflow tract (RVOT) in patients with BrS who were undergoing open chest surgery^24^. This implies that the characteristic monophasic AP morphology recorded in a patient with BrS might be attributed to the selective decrease of *I*_Na,LM_ in epicardial myocytes located in the RVOT.

In epicardial myocytes in the RVOT, the loss-of-dome AP followed by marked shortening of APD, i.e., repolarization abnormality^7,26^, is considered to be one of the mechanisms of coved-type ST-segment elevation (Brugada-type ECG)^2,6^. One of the factors causing the loss-of-dome is thought to be the ventricular gradient of *I*_to_ through the RV wall, as well as endogenously heterogeneous *I*_to_ in the RV epicardial layers^7,26^. We showed that even without changing *I*_to_, the loss-of-dome type abbreviated AP was also caused by extreme decrease and/or loss of Na^+^ channels from the LM, but not JMs of each myocyte (Fig. 2A*d*,B; green lines). Indeed, the loss of *I*_Na,LM_ further decreased the AP phase-0 amplitude, therefore preventing the *V*_m_ depolarization at AP phase-0 from reaching a threshold potential for *I*_CaL_ activation; this thus failed to activate *I*_CaL_, resulting in the loss-of-dome type abbreviated AP. These results suggest that the Na^+^ channel abnormality, which is commonly believed to be the cause of the depolarization abnormality in BrS^26^, may also be responsible for the repolarization abnormality of BrS.

We found that the conduction delay in RVOT under structurally normal hearts in patients with BrS^27^, i.e., depolarization disorder hypothesis^26^, may be attributable to the selective decrease in *I*_Na,LM_ causing decreases in *I*_g_ without the *G*_g_ change. The mechanism of *I*_Na,LM_-mediated decreases in *I*_g_ proposed in the present theoretical study is as follows: (1) The marked decrease in *I*_Na,LM_ decreases the pre-JM current (compare left to right panels of Fig. 2C*c*) and thus slows the depolarization of the pre-JM (compare left to right panels of Fig. 2C*e*). (2) The slower and smaller depolarization of the pre-JM due to the reduced pre-JM current leads to decreases in the difference between ${V}_{\text{i},\text{pre-JM}}^{k}$ and ${V}_{\text{i},\text{post-JM}}^{k+1}$, for *k* = 1, 2,…, 299, resulting in the *I*_g_ decrease. This notion is also supported by previous experimental studies^14,15^, which showed that mutant mice hearts with a selective decrease in Na^+^ channel expression on the LM had slower CV than the wild type, without affecting the connexin 43 (Cx43; gap-junction channel-forming proteins) expression (see Supplementary Table S1).

The underlying mechanism of P2R has been explained by the electrotonic interaction between the region with normal AP morphology and the adjacent region with the loss of the AP dome. Such P2R mechanism were explored in numerous experimental^28,29^ and computational studies^30–32^. For the onset of P2R, excitable tissues such as RV and RVOT, in general, are required to be separated into two regions by the segment of reduced excitability and/or reduced electrical coupling. The existence of a local difference in AP waveform (or local APD difference) at the RVOT epicardium has been confirmed by several experimental studies^9,33^. Our results showed that an existence of the slight spatial heterogeneity at tissue-level in Na^+^ channel expressions accompanied by a marked decrease in *I*_Na,LM_ (reduced excitability) could induce P2R (Fig. 3). Although the causes that make the local differences of APs in the RVOT/RV are not clear, one possible cause is an endogenous heterogeneity in Na^+^ channel expression at the RVOT epicardial layer as in the transmural difference in Na^+^ channel expression in the RVOT and the RV free wall^34^. On the other hand, Auerbach and co-workers^35^ have shown by combined experimental and computational studies that a structural heterogeneity (a geometric expansion) can produce P2R. In addition to differences in electrophysiological properties due to differences in the developmental origin of the RVOT and RV^36^, a tissue structure of the RVOT with more aligned fiber orientations is different from that of the RV free wall which comprised of network-like structures of myocardial fibers; the structural heterogeneity between the RV free wall and RVOT might be also responsible for the P2R development in patients with BrS.

A previous clinical observation^37^ has demonstrated that BrS is associated with epicardial interstitial fibrosis and reduced Cx43 expression in the RVOT. Furthermore, previous experimental studies using the *SCN5A* heterozygous (*Scn5a*+/−) mice^38,39^ have shown that fibrosis, accompanied by downregulation and redistribution of Cx43, is increased with aging. The *I*_g_ decrease with *G*_g_ change leads to a decrease in the local current (*I*_m,LM_) consisting of *I*_g_ and *I*_m,post-JM_ (Fig. 2C*b*,*g*). Besides *I*_Na,LM_ reduction, decreases in *I*_g_ could result in the loss-of-dome abbreviated AP^32^. Therefore, we speculate that the decrease in gap junctions at the RVOT, where Na^+^ channel expression is impaired along with aging-associated fibrosis, may also cause the loss of the AP dome followed by P2R resulting in VT/VF.

Miyoshi et al.^31^ suggested that *I*_CaL_ plays critical roles in the formation of local APD differences even during AP propagation and the following second excitation during P2R. Indeed, as shown in Figs. 2B and 4B, *I*_CaL_ was the main determinant of AP dome formation in each myocyte of the myocardial strand and contributed to maintaining the second excitation wave propagation during P2R, consequently leading to reentrant excitation wave propagations (VT/VF) in a myocardial ring model (Fig. 6). Notably, the reentrant waves shown in Fig. 6B*b* combined slow (*I*_CaL_ based AP phase-2 dome propagation) and fast (*I*_Na_ and intercellular local current based loss-of-dome AP propagation) wavefronts, and are likely to share a similar mechanism with the bi-stable wave propagation reported by Chang et al.^40^ On the other hand, it is known that the sympathetic activation in patients with BrS decreases VT/VF initiation^41^, predicting that the greater *I*_CaL_ enhancement contributes to preventing P2R. We demonstrated that β-AS by the administration of isoproterenol could reduce the local APD difference and could prevent P2R (Fig. 5). Based on our result as shown in Fig. 4, *I*_CaL_ inhibition by Ca^2+^ channel blocker and/or β-blocker administration is likely to prevent the P2R development (Supplementary Fig. S5), consequently preventing VT/VF. However, the effects of β-AS and/or β-blockers on AP propagation in patient with BrS are not fully understood. Thus, more quantitative experimental verification and theoretical studies using more elaborate intracellular Ca^2+^-signaling models^42^ are required to understand the exact roles of *I*_CaL_ modulation by β-AS and/or β-blocker in P2R and arrhythmia preventions.

Based on our simulation data (Supplemental Fig. S4), it is suggested that the ephaptic coupling mechanism is essential for the loss of AP dome followed by P2R development. Such ephaptic coupling might be involved in several heart diseases. Tolkacheva and co-workers have investigated the role of Na^+^ channel distribution on AP conduction during regional ischemia^17,43^. They observed small APs with a reduced amplitude that propagates and impacts conduction failure. In patients with long QT syndrome type 3, the distribution changes in Na^+^ channels with the gain-of-function mutation have significantly affected the propagation in APs with early afterdepolarizations^44–46^. Also Jaeger et al.^47^ have demonstrated a mechanism of conduction failure due to changes in Na^+^ channel distribution. As with these, we will need to perform additional simulations employing more sophisticated, three-dimensional, whole ventricle models to elucidate the development mechanism of severe arrhythmia disorders such as BrS.

## Methods

### Myocardial strand and ring models and subcellular Na^+^ channel distribution

To perform computer simulations examining effects of alterations in subcellular Na^+^ channel distribution on AP propagation behaviors, we constructed myocardial strand and ring models comprising 300 (Fig. 1B*a*,*d*) and 600 ventricular myocytes (Fig. 1C), respectively. As shown in Fig. 1B*b*, adjacent myocytes were electrically coupled with both gap junctions and an electric field mechanism (ephaptic coupling)^48,49^, the latter of which is an electrical interference effect caused at intercellular cleft space; electrical communication between myocytes through the electric field mechanism is mediated by large negative changes in the extracellular potential elicited within the narrow intercellular cleft space facing the IDs.

As in our previous study^13,16^, to achieve the inhomogeneous Na^+^ channel distribution within a myocyte found in humans and other mammals^50,51^, the whole cell membrane of each myocyte was divided into three segments (Fig. 1B*c*), one segment for the LM and the other two segments for the pre- and post-junctional membranes (JMs), namely the IDs. Allocating Na^+^ channel conductances to each membrane segment and changing those individually, we altered the subcellular expression of Na^+^ channels. The electrophysiological property of each membrane segment was represented by a modified O’Hara-Rudy dynamic (mORd) model^10,11^, which is the most sophisticated human ventricular AP model to date that extensively validated against experimental data from more than 100 non-diseased human hearts. For better correspondence with the experimental CV of human RV epicardium^52^, the formula of fast *I*_Na_ in the O’Hara-Rudy dynamic model was replaced with that of the ten Tusscher-Panfilov (TP) model^12^. We set the control conductance of fast *I*_Na_ to 11 nS/pF for the LM (*G*_NaF,LM_) and 44 nS/pF for the JMs (*G*_NaF,JM_) so that *I*_Na_ amplitude obtained from the simulation matched the one recorded experimentally for basal condition^15^; the conductance of late *I*_Na_ was also set to 0.0045 nS/pF for the LM (*G*_NaL,LM_) and 0.018 nS/pF for the JMs (*G*_NaL,JM_) as the control condition. The fast and late Na^+^ channel conductances of the JM and LM were expressed as percentages of the *G*_NaF,JM_ (*G*_NaL,JM_) and *G*_NaF,LM_ (*G*_NaL,LM_), i.e., %*g*_Na,JM_ and %*g*_Na,LM_, respectively. Specific parameters in the myocardial strand and ring models consisting of the mORd model can be found in the Supplementary Table S2.

To test whether the sympathetic activity inhibits the P2R development, we additionally performed simulations of AP propagation using the myocardial strand model under the condition of β-adrenergic stimulation (β-AS) mimicking sympathetic activation. Modifications of parameters for simulating the condition of β-AS^18,19^ were listed in the Supplementary Table S3.

### Simulations

Numerical simulations of AP propagation were performed as described previously^13,16^ and details were provided in Supplementary Methods. Pacing stimuli consisting of 300 pA/pF, 1 ms current pulse with a basic cycle length of 1 s were applied to a myocyte located at the end of the myocardial strand (the 300th myocyte in the myocardial ring) and repeated 30 times to minimize transient responses in each simulation.

### Preprint

A previous version of this manuscript was published as a preprint^53^.

## Supplementary information

Supplementary Information.

## Data Availability

All data generated or analyzed during this study are included in this published article (and its Supplementary Information files). Furthermore, these codes used to simulate the myocardial strand and ring models are available in the repository: https://github.com/92tsumoto/BrS-P2R-strand-ORd2011model-withTNNP_INa-FT_IKr, and https://github.com/92tsumoto/BrS-P2R-ring-ORd2011model-withTNNP_INa-FT_IKr.
