# Supplementary material for: Specific decreasing of Na+ channel expression on the lateral membrane of cardiomyocytes causes fatal arrhythmias in Brugada syndrome
Source: Sci Rep. 2020 Nov 17;10:19964. doi: 10.1038/s41598-020-76681-3 (PMC7673036; doi:10.1038/s41598-020-76681-3)
Supplement: Supplementary file 1 — Supplementary Information. [file 41598_2020_76681_MOESM1_ESM.pdf]

Supplementary Information

Specific decreasing of Na^+ channel expression on the lateral membrane of cardiomyocytes causes fatal arrhythmias in Brugada syndrome

Kunichika Tsumoto[1,2,*], Takashi Ashihara[3], Narumi Naito[4], Takao Shimamoto[4], Akira Amano[4], Yasutaka Kurata[1], Yoshihisa Kurachi[2,5]

[1]Department of Physiology, Kanazawa Medical University, Uchinada, 920-0293, Japan
[2]Department of Pharmacology, Graduate School of Medicine, Osaka University, Suita, 565-0871, Japan
[3]Department of Medical Informatics and Biomedical Engineering, Shiga University of Medical Science, Otsu, 520-2192, Japan
[4]Department of Bioinformatics, College of Life Sciences, Ritsumeikan University, Kusatsu, 525-8577, Japan
[5]Glocal Center for Medical Engineering and Informatics, Osaka University, Suita, 565-0871, Japan

Corresponding author: Kunichika Tsumoto
Email: tsumoto@kanazawa-med.ac.jp

This PDF file includes:

Tables S1 to S3
Supplementary Methods
Supplementary References
Supplementary Figures S1 to S5

Table S1. Comparison with experimental results in ΔSIV mice

		100%$g_{Na,LM}$ (WT)	35%$g_{Na,LM}$ (ΔSIV)	
Peak $I_{Na,LM}$ (pA/pF)	Tsumoto et al	329.3	138.7	57.9 % ↓
	Shy et al (12)	301.1 ± 25.4	115.0 ± 11.9	61.8 ± 4.0 % ↓
CV (cm/s)	Tsumoto et al	71.4	53.6	24.9 % ↓
	Shy et al (12)	70.1 ± 4.4	54.2 ± 2.9	22.7 ± 0.7 % ↓

Tsumoto et al, Present Study; Shy et al, (12)

Table S2. Control parameter values

Parameter	Definition	value
G_i	Cytosolic conductance	2.327 μS
G_g	Intercellular gap junctional conductance	2.327 μS
G_j	Radial cleft conductance	0.25 μS
G_d	Axial cleft conductance	14.0 mS
$C_{m,pre\text{-}JM}$, $C_{m,post\text{-}JM}$	Capacitance of the pre- and post-junctional membranes	5.8 pF
$C_{m,LM}$	Capacitance of the lateral membrane	173.4 pF
$G_{NaF,pre\text{-}JM}$, $G_{NaF,post\text{-}JM}$	Maximum conductance density of fast Na^+ channel of the pre- and post-junctional membranes	44 nS/pF
$G_{NaF,LM}$	Maximum conductance density of fast Na^+ channel of the lateral membrane	11 nS/pF
$G_{NaL,pre\text{-}JM}$, $G_{NaL,post\text{-}JM}$	Maximum conductance density of late Na^+ channel of the pre- and post-junctional membranes	0.03 nS/pF
$G_{NaL,LM}$	Maximum conductance density of late Na^+ channel of the lateral membrane	0.0075 nS/pF
G_{kr}	Maximum conductance density of rapidly activating delayed rectifier K^+ channel	0.058 nS/pF
P_{Ca}	Ca^{2+} permeability for the L-type Ca^{2+} channel	0.00013 cm/s

JM, junctional membrane; LM, lateral membrane

Table S3. Modification parameters for the condition of β-adrenergic stimulation (β-AS)

Parameter	Control	β-AS (×ratio)
Ca^{2+} permeability for the L-type Ca^{2+} channel (P_{Ca})	0.00013 cm/s	×3.0
Maximum conductance (G_{Ks}) of slowly activating delayed rectifier K^+ channel current (I_{Ks})	0.00476 nS/pF	×2.0
Maximum Na^+-K^+ pump current (I_{NaK})	27 pA/pF	×1.2
Maximum rate of Ca^{2+} uptake to SR	1.3 mM/ms	×1.41
Half-activation voltage in I_{Ks} activation gate	−11.6 mV	−19.6 mV

Supplementary Methods

Myocardial strand and ring models

We constructed myocardial strand and ring models comprising 300 (Fig. 1B*a*) and 600 ventricular myocytes (Fig. 1C), respectively. The cytosolic conductance (G_i) of each myocyte was 2.327 μS, calculated from $G_i = \sigma_{myo} \cdot \pi \cdot r^2 / l$ (1-4), where σ_{myo} (11.1 mS/cm) is the cytosolic conductivity, and l (150 μm) and r (10 μm) are the length and radius of the myocyte, respectively (5, 6).

As it was well-known, the intercalated discs (IDs) are very complex morphology, and the intercellular cleft (intercellular junction) facing the IDs is extremely tight space with tortuous structures. Such complex tight space could significantly interfere with the cation movement such as Na^+, K^+, and Ca^{2+}. Thus extracellular conductivity (σ_{ext}) in the intercellular junction may be lower than the σ_{myo}. In the present study, we set the extracellular conductivity (σ_{ext}) in the cleft space to 6.7 mS/cm. Then, the radial cleft conductance (G_j) and series axial cleft conductance (G_d) were defined as functions of the cleft width (cw), $G_j = 8 \cdot \pi \cdot cw \cdot \sigma_{ext}$ and $G_d = \sigma_{ext} \cdot \pi \cdot r^2 / cw$, respectively (1-4). The cw, which is the distance between the pre- (pre-JM) and post-junctional membrane (post-JM), has been estimated to be 20−100 nm from the literatures (7-9). However, its width may not be uniform at the intercellular junction. The cleft space exists a narrow stretch of closely apposed membranes (5−30 nm), which referred to as perinexus, located immediately adjacent to gap-junctions, and the perinexus may facilitate ephaptic conduction. Therefore, we set cw to 15 nm as an averaged value with reference to the previous reports (1-3).

Electrophysiological properties of each membrane segment composing the ventricular myocyte were described by a modified O'Hara–Rudy dynamic (mORd) model (10). The mORd model consisted of a membrane capacitance and several ion channel currents, including our modification (11) to the original currents of a potassium channel (I_{Kr}). From experimental data (5, 6), the entire membrane capacitance of a human ventricular myocyte was set to 185 pF ($C_{m,post\text{-}JM} = C_{m,pre\text{-}JM}$ = 5.8 pF; $C_{m,LM}$ = 173.4 pF) and the initial value of $[Na^+]_i$ was set to 7.0 mM. Tables S2 and S3 show the modification parameters of the relevant models.

Calculations

The calculation methods for action potential propagation in the myocardial strand and ring models are as follows. From the equivalent circuits of the myocyte model shown in Fig. 1B*d*, let us give the following current vector (**I**) comprised of the transmembrane current from each node toward each membrane segment:

$$\mathbf{I} = \left[i_{m,1}^{1} \quad i_{m,2}^{1} \quad \cdots \quad i_{m,3}^{k-1} \quad \middle| i_{m,1}^{k} \quad i_{m,2}^{k} \quad i_{m,3}^{k} \middle| \; i_{m,1}^{k+1} \quad \cdots \quad i_{m,2}^{N} \quad i_{m,3}^{N} \right]^T, \tag{S1}$$

where $[\ ]^T$ represents the transpose operation and N is the total myocyte number. Furthermore, we suppose that a vector (V) consisting of the functions of the transmembrane potential of each membrane segment is given by

$$\mathbf{V} = \left[v_{1,1} \quad v_{1,2} \quad \cdots \quad v_{k-1,3} \quad \middle| v_{k,1} \quad v_{k,2} \quad v_{k,3} \middle| \; v_{k+1,1} \quad \cdots \quad v_{N,2} \quad v_{N,3} \right]^T. \tag{S2}$$

Then, each function, $v_{k,l}$, for k = 1, …, N and l = 1, 2, and 3, except for the first function ($v_{1,1}$) and the final one ($v_{N,3}$), in the voltage vector is given by

$$\begin{aligned} v_{k,1} &= 2G_i\left(V_{\mathrm{m,post-JM}}^{k} - V_{\mathrm{m,LM}}^{k}\right) - G_g\left(V_{\mathrm{m,pre-JM}}^{k-1} - V_{\mathrm{m,post-JM}}^{k}\right) \\ v_{k,2} &= -2G_i\left(V_{\mathrm{m,post-JM}}^{k} - 2V_{\mathrm{m,LM}}^{k} + V_{\mathrm{m,pre-JM}}^{k}\right) \\ v_{k,3} &= -2G_i\left(V_{\mathrm{m,LM}}^{k} - V_{\mathrm{m,pre-JM}}^{k}\right) + G_g\left(V_{\mathrm{m,pre-JM}}^{k} - V_{\mathrm{m,post-JM}}^{k+1}\right). \end{aligned} \tag{S3}$$

In cases of k = 1 and k = N, $v_{1,1}$ and $v_{N,3}$ are provided by $v_{1,1} = 2G_i\left(V_{\mathrm{m,post-JM}}^{1} - V_{\mathrm{m,LM}}^{1}\right)$ and $v_{N,3} = -2G_i\left(V_{\mathrm{m,LM}}^{N} - V_{\mathrm{m,pre-JM}}^{N}\right)$, respectively. Using the current vector (Eq. S1) and the vector (Eq. S2), we can derive a linear equation at arbitrary time t using Ohm's law and Kirchhoff's law, leading to the following simultaneous equation:

$$\mathbf{RI}=\mathbf{V}, \tag{S4}$$

where $\mathbf{R}$ is the $3N \times 3N$ matrix. The matrix, $\mathbf{R}$, in Eq. S4 is given by Eq. S5:

$$\mathbf{R} = \left[\begin{array}{ccc|ccc|ccc|ccc|ccc}
-1 & & & & & & & & & & & & & & \\
 & -1 & p_1 & p_2 & & & & \mathbf{0} & & & \mathbf{0} & & & \mathbf{0} & \\
 & & p_3 & p_4 & & & & & & & & & & & \\
\hline
 & & p_4 & p_3 & & & & & & & & & & & \\
 & & & & \ddots & & & & & & \mathbf{0} & & & \mathbf{0} & \\
 & & & & & p_3 & p_4 & & & & & & & & \\
\hline
 & & & & & p_4 & p_3 & & & & & & & & \\
 & \mathbf{0} & & & & p_2 & p_1 & -1 & p_1 & p_2 & & & & \mathbf{0} & \\
 & & & & & & & & p_3 & p_4 & & & & & \\
\hline
 & & & & & & & & p_4 & p_3 & & & & & \\
 & \mathbf{0} & & & \mathbf{0} & & & & & & \ddots & & & & \\
 & & & & & & & & & & & p_3 & p_4 & & \\
\hline
 & & & & & & & & & & & p_4 & p_3 & & \\
 & \mathbf{0} & & & \mathbf{0} & & & \mathbf{0} & & & & p_2 & p_1 & -1 & \\
 & & & & & & & & & & & & & & -1
\end{array}\right], \tag{S5}$$

where $p_1 = 2G_i/G_j + G_i/G_d$, $p_2 = 2G_i/G_j$, $p_3 = -1.0 - 2G_i/G_j - G_i/G_d - 0.5G_g/G_d$, and $p_4 = -2G_i/G_j + 0.5G_g/G_d$. Equation S4 can be solved for the current *I* with $V_{\mathrm{m},l}^{k}$, for l = post-JM, LM, and pre-JM, at time *t* as an initial condition. Furthermore, in the case of the myocardial ring model, the following matrix was employed:

$$\mathbf{R} = \left[\begin{array}{ccc|ccc|ccc|ccc|ccc}
p_3 & & & & & & & & & & & & & & p_4 \\
p_1 & -1 & p_1 & p_2 & & & & \mathbf{0} & & & \mathbf{0} & & & & p_2 \\
 & & p_3 & p_4 & & & & & & & & & & & \\
\hline
 & & p_4 & p_3 & & & & & & & & & & & \\
 & & & & \ddots & & & & & & \mathbf{0} & & & \mathbf{0} & \\
 & & & & & p_3 & p_4 & & & & & & & & \\
\hline
 & & & & & p_4 & p_3 & & & & & & & & \\
 & \mathbf{0} & & & & p_2 & p_1 & -1 & p_1 & p_2 & & & & \mathbf{0} & \\
 & & & & & & & & p_3 & p_4 & & & & & \\
\hline
 & & & & & & & & p_4 & p_3 & & & & & \\
 & \mathbf{0} & & & \mathbf{0} & & & & & & \ddots & & & & \\
 & & & & & & & & & & & p_3 & p_4 & & \\
\hline
 & & & & & & & & & & & p_4 & p_3 & & \\
p_2 & & & & \mathbf{0} & & & \mathbf{0} & & & & p_2 & p_1 & -1 & p_1 \\
p_4 & & & & & & & & & & & & & & p_3
\end{array}\right]. \tag{S6}$$

The transmembrane potential in each segment is given by

$$C_{\mathrm{m},l}\frac{\mathrm{d}V_{\mathrm{m},l}^{k}}{\mathrm{d}t} + I_{\mathrm{ion}}\left(V_{\mathrm{m},l}^{k}, t\right) = i_{\mathrm{m},l}^{k}, \tag{S7}$$

for k = 1, …, N and l = post-JM, LM, and pre-JM, where $C_{\mathrm{m},l}$ (pF) is the membrane capacitance in each membrane segment, I_{ion} (μA/μF) is the sum of several ion channel currents in the mORd model, $i_{\mathrm{m},l}^{k}$ (μA/μF) is the transmembrane current corresponding to each element in the current vector defined by Eq. S1. We should note that $i_{\mathrm{m,post-JM}}^{k}$, $i_{\mathrm{m,LM}}^{k}$, and $i_{\mathrm{m,pre-JM}}^{k}$ are equal to $i_{\mathrm{m},1}^{k}$, $i_{\mathrm{m},2}^{k}$, and $i_{\mathrm{m},3}^{k}$, respectively. For an arbitrary time *t*, all the membrane currents, $i_{\mathrm{m},l}^{k}$, are obtained by solving Eq. S4 with the transmembrane potentials as an initial condition. Thus, we calculated all

transmembrane potentials at time $t + \Delta t$ in each segment, where Δt corresponds to the time step in the Euler method. The time step, Δt, was set to 1 μs. These codes used to simulate the myocardial strand and ring models are available in the repository: https://github.com/92tsumoto/BrS-P2R-strand-ORd2011model-withTNNP_INa-FT_IKr, and https://github.com/92tsumoto/BrS-P2R-ring-ORd2011model-withTNNP_INa-FT_IKr

Supplementary References

1. Tsumoto, K., Ashihara, T., Haraguchi, R., Nakazawa, K. & Kurachi, Y. Ischemia-related subcellular redistribution of sodium channels enhances the proarrhythmic effect of class I antiarrhythmic drugs: a simulation study. *PLoS One.* **9**(10), e109271; 10.1371/journal.pone.0109271 (2014).

2. Tsumoto, K., Ashihara, T., Haraguchi, R., Nakazawa, K. & Kurachi, Y. Roles of subcellular Na^+ channel distributions in the mechanism of cardiac conduction. *Biophys J*. **100**(3), 554−563 (2011).

3. Kucera, J. P., Rohr, S. & Rudy, Y. Localization of sodium channels in intercalated disks modulates cardiac conduction. *Circ Res*. **91**(12), 1176−1182 (2002).

4. Mori, Y., Fishman, G. I. & Peskin, C. S. Ephaptic conduction in a cardiac strand model with 3D electrodiffusion. *Proc Natl Acad Sci U S A*. **105**(17), 6463−6468 (2008).

5. Virág, L. *et al*. The slow component of the delayed rectifier potassium current in undiseased human ventricular myocytes. *Cardiovasc Res.* **49**(4), 790−797 (2001).
6. Iost, N. *et al*. Delayed rectifier potassium current in undiseased human ventricular myocytes. *Cardiovasc Res*. **40**(3), 508−515 (1998).
7. Hoagland, D. T., Santos, W., Poelzing, S. & Gourdie, R. G. The role of the gap junction perinexus in cardiac conduction: Potential as a novel anti-arrhythmic drug target. *Prog Biophys Mol Biol.* **144**, 41−50; 10.1016/j.pbiomolbio.2018.08.003 (2019).
8. Veeraraghavan, R., Lin, J., Keener, J. P., Gourdie, R, & Poelzing, S. Potassium channels in the Cx43 gap junction perinexus modulate ephaptic coupling: an experimental and modeling study. *Pflugers Arch.* **468**(10), 1651−1661; 10.1007/s00424-016-1861-2 (2016).
9. Veeraraghavan, R., Lin, J., Hoeker, G. S., Keener, J. P., Gourdie, R. G., & Poelzing, S. Sodium channels in the Cx43 gap junction perinexus may constitute a cardiac ephapse: an experimental and modeling study. *Pflugers Arch*. **467**(10), 2093−105; 10.1007/s00424-014-1675-z (2015).
10. O'Hara, T., Virág, L., Varró, A. & Rudy, Y. Simulation of the undiseased human cardiac ventricular action potential: model formulation and experimental validation. *PLoS Comput Biol*. **7**(5) e1002061; 10.1371/journal.pcbi.1002061 (2011).

11. Furutani, K. *et al*. Facilitation of I_{Kr} current by some hERG channel blockers suppresses early afterdepolarizations. *J Gen Physiol*. **151**(2), 214−230 (2019).

12. Shy, D. *et al*. PDZ domain-binding motif regulates cardiomyocyte compartment-specific $Na_V1.5$ channel expression and function. *Circulation*. **130**(2), 147−160 (2014).

Supplementary Figure Legends

Figure S1: Effects of changing the Na^+ channel conductance in the junctional membrane (JM) on action potential (AP) morphology and conduction velocity (CV).
A, AP propagation observed in the myocardial strand model when the Na^+ channel conductance on the JMs ($g_{Na,JM}$) was selectively reduced to 10% (*a*), 5% (*b*), 3% (*c*), and 0% (*d*). CVs at 10%$g_{Na,JM}$, 5%$g_{Na,JM}$, 3%$g_{Na,JM}$ and 0%$g_{Na,JM}$ were all 68.2 cm/s. **B**, The post-junctional ($I_{m,post\text{-}JM}$) and pre-junctional ($I_{m,pre\text{-}JM}$) transmembrane currents (*a*), the transmembrane current ($I_{m,LM}$) and I_{Na} ($I_{Na,LM}$) in the lateral membrane (LM) segment (*b*), the junctional membrane I_{Na} ($I_{Na,JM}$) of the pre-junctional membrane (pre-JM) in the 150th myocyte and of the post-junctional membrane (post-JM) in the 151st myocyte (*c*), the extracellular cleft potential (V_j) between the 150th and 151st myocytes (*d*), the intracellular potential (V_i) of the LM and pre-JM in the 150th myocyte and of the post-JM in the 151st myocyte (*e*), and the gap junctional current (I_g) between the 150th and 151st myocytes (*f*).

Figure S2: Effects of decreasing Na^+ channels on both the lateral membrane (LM) and junctional membranes (JMs) on action potential (AP) propagation.
A, AP propagation observed in the myocardial strand model when the total Na^+ channel conductance ($g_{Na,tot}$) was uniformly reduced to 50% (*a*), 25% (*b*), 13% (*c*), and 10% (*d*). Conduction velocities at 50%$g_{Na,tot}$, 25%$g_{Na,tot}$, 13%$g_{Na,tot}$ and 10%$g_{Na,tot}$ were 55.6, 40.5, 25.9, and 17.4 cm/s, respectively. **B**, The membrane potential (V_m) of LM in the 150th myocyte (*a*), the post-junctional ($I_{m,post\text{-}JM}$) and pre-junctional ($I_{m,pre\text{-}JM}$) transmembrane currents (*b*), the transmembrane current ($I_{m,LM}$) and I_{Na} ($I_{Na,LM}$) in the LM segment (*c*), the junctional membrane I_{Na} ($I_{Na,JM}$) of pre-junctional membrane (post-JM) in the 150th myocyte and of post-junctional membrane (post-JM) in the 151st myocyte (*d*), the extracellular cleft potential (V_j) between the 150th and 151st myocytes (*e*), and the gap junctional current (I_g) between the 150th and 151st myocytes (*f*).

Figure S3: Ionic mechanism of action potential (AP) alternans.
Time-dependent behaviours of the membrane potential V_m (**A**), the activation gating variable of the Na^+ channel, m (**B**), the inactivation variable as a product of the fast (h) and slow (j) inactivation gating variables, $h \times j$ (**C**), I_{Na} (**D**), and L-type Ca^{2+} channel current, I_{CaL} (**E**) in the lateral membrane (LM) segment of the 150th cell in the myocardial strand model under the same condition as for Fig. 3A*a* (6%$g_{Na,LM}$ in the proximal and 7%$g_{Na,LM}$ in the distal). Blue and green traces show the changes in V_m, m, and $h \times j$, $I_{Na,LM}$, and I_{CaL} during AP propagation in response to the 29th and 30th stimuli, respectively. Each panel in the right column (**C**-**E**) shows the enlargement of the area indicated by the boxes in each corresponding panel in the left column.

Figure S4: Effects of changes in the cleft width (*cw*) on action potential (AP) propagation.
AP propagations in the myocardial strand model in the case of *cw* = 7 nm (**A**), 17 nm (**B**), 20 nm (**C**), and infinity (i.e., no ephaptic coupling) (**D**). Based on the results shown in Fig. 3B for the condition that evoked the robust P2R, the Na^+ channel conductances on LM in the proximal (cell #1-200) and the distal (cell #201-300) were set to 4% and 10%, respectively. Conduction velocities (CVs) in the proximal part of the myocardial strand with *cw* = 7 nm (**A**), 17 nm (**B**), 20 nm (**C**), and without ephaptic coupling (**D**) were 20.5, 31.3, 33.3, and 40.5 cm/s, respectively. In addition, CVs in the distal part of the strands with *cw* = 7 nm, 17 nm, 20 nm, and without ephaptic coupling were 30.6, 37.5, 38.5, and 45.5 cm/s, respectively. Simulated AP propagation (*a*), simulated behaviors of the membrane potential (V_m) in several myocytes (#100-220) (*b*), the extracellular cleft potential, V_j (*c*), the gap junctional current, I_g, flowing from the cell #100 to #220 (*d*), I_{Na} in the post-junctional membrane ($I_{Na,post\text{-}JM}$) (*e*), I_{Na} in the lateral membrane ($I_{Na,LM}$) (*f*), and I_{Na} in the pre-junctional membrane ($I_{Na,pre\text{-}JM}$) (*g*).

Figure S5: A potential preventing effect of I_{CaL} blockers on phase-2 reentry.
A simulated action potential propagation in the myocardial strand model under the same condition as for Fig. 4 but during 50% inhibition of I_{CaL}. The Na^+ channel conductances on the lateral

membrane were set to 4% and 7% in the proximal (cell #1-200) and distal (cell #201-300), respectively, while keeping the Na^+ channel conductance on junctional membranes at the control value.

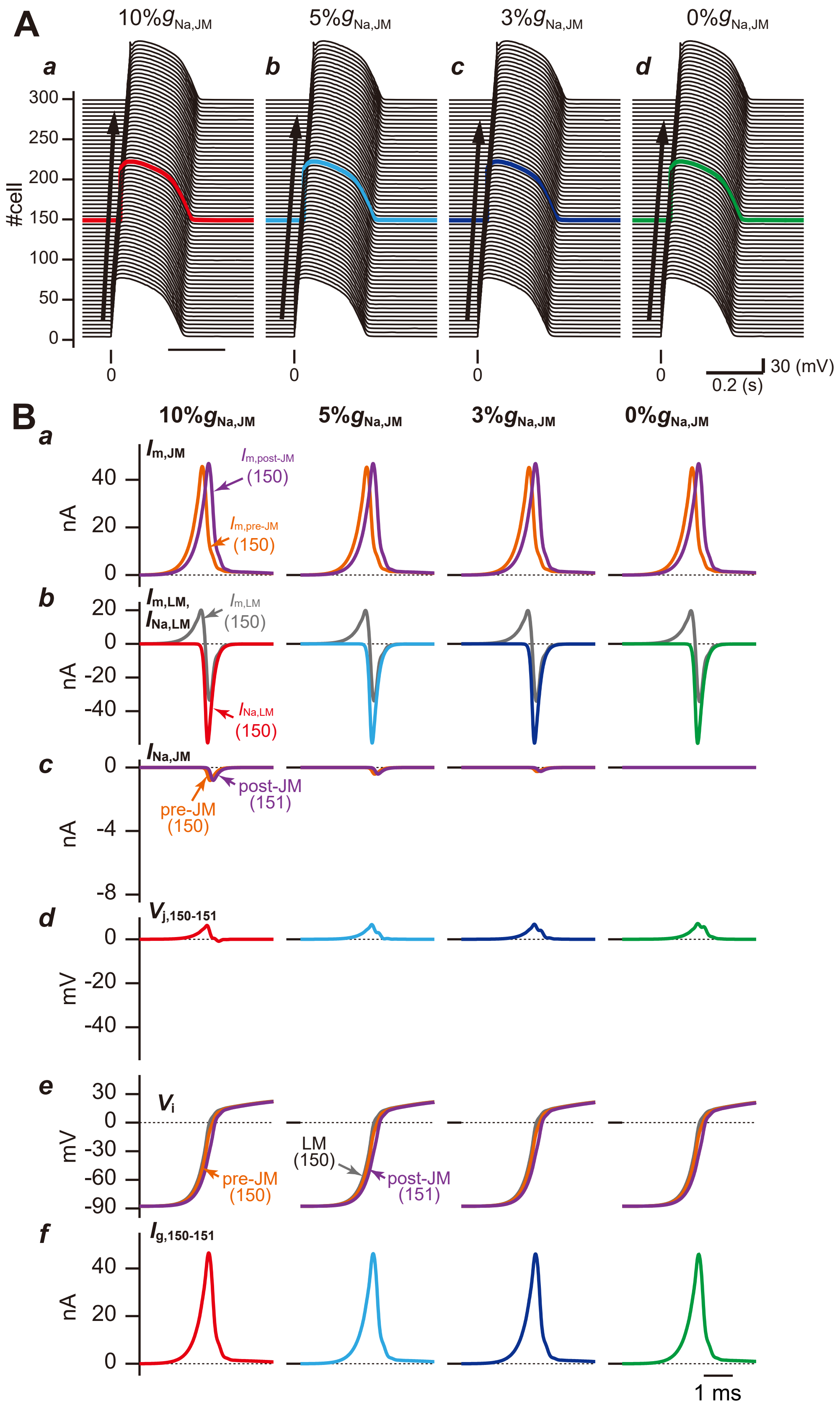

Supplementary Figure S1

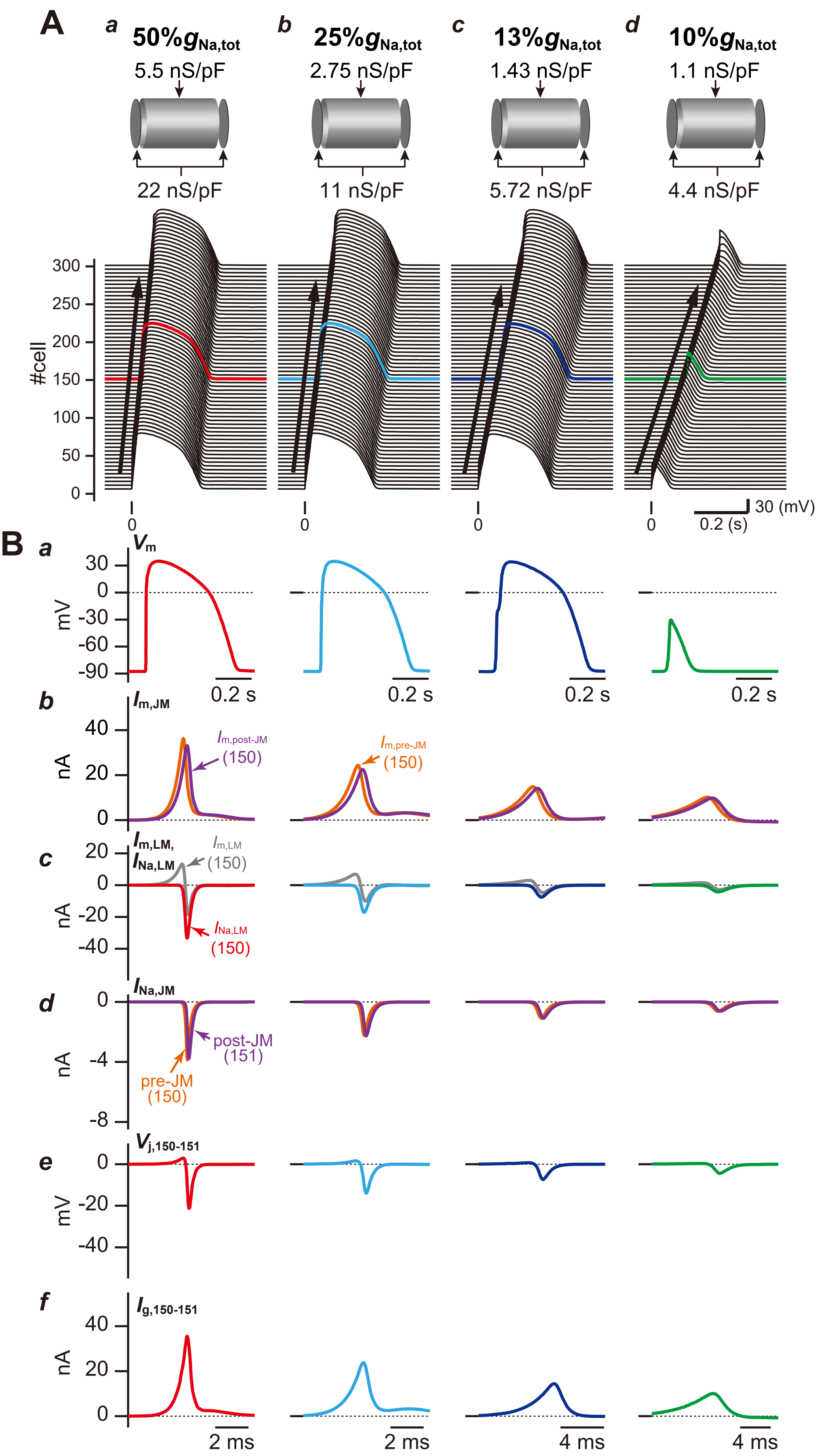

Supplementary Figure S2

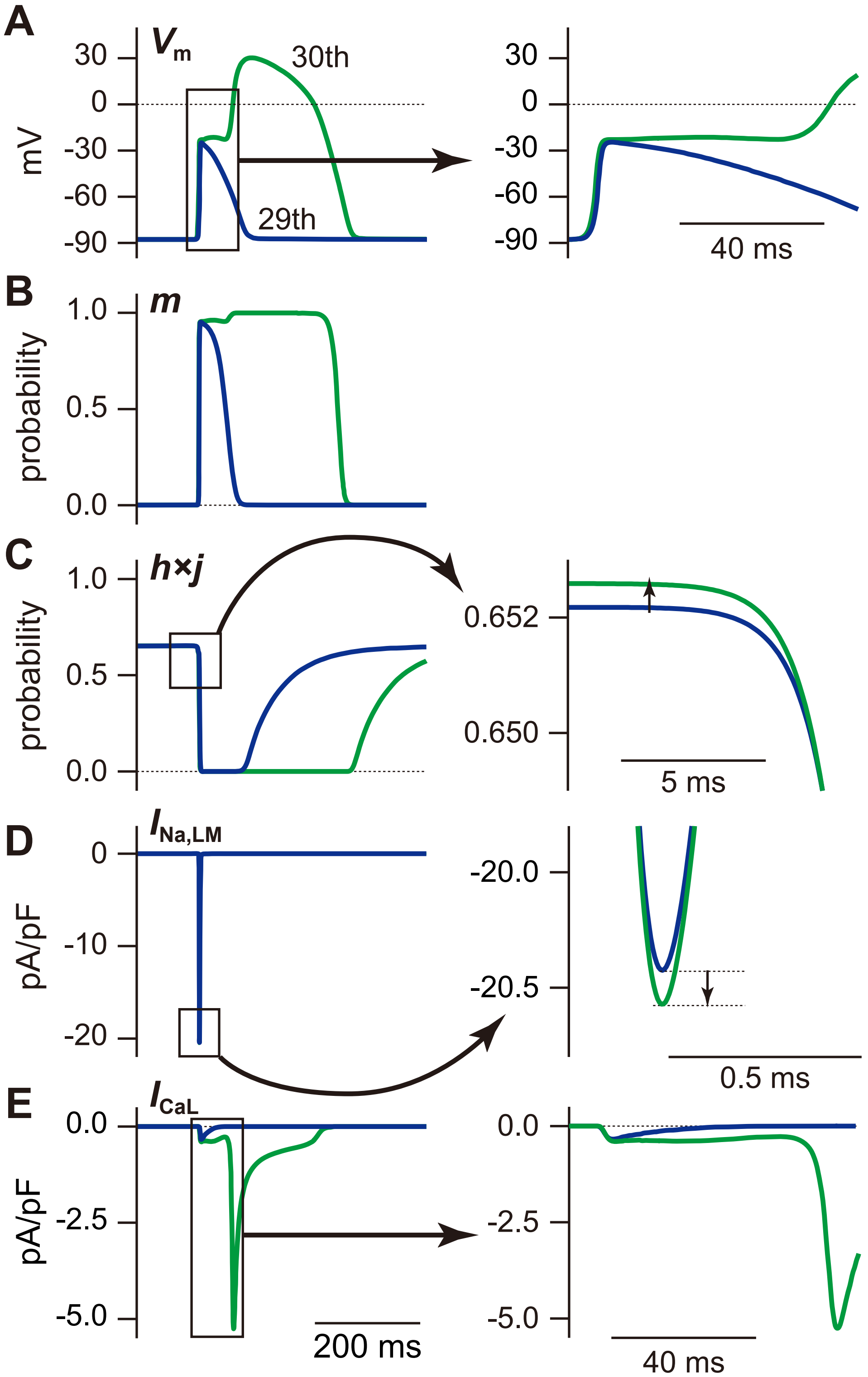

Supplementary Figure S3

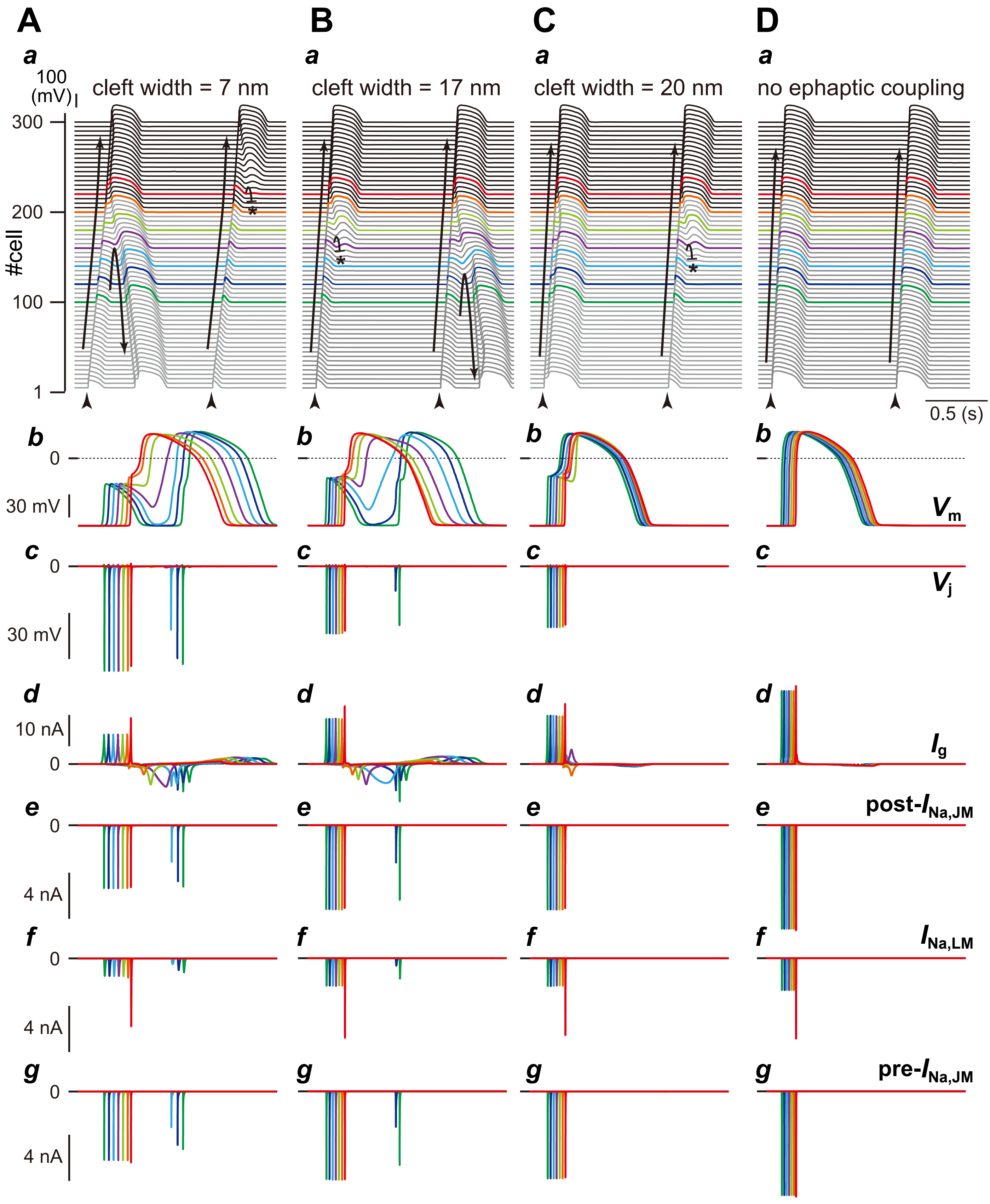

Supplementary Figure S4

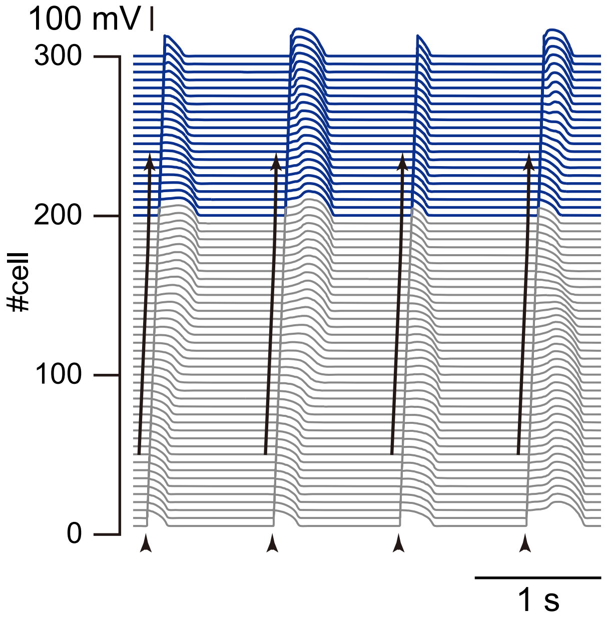

Supplementary Figure 5
